# Impact of Smoking, Body Weight, Diabetes, Hypertension and Kidney Dysfunction on Survival in Pancreatic Cancer Patients—A Single Center Analysis of 2323 Patients within the Last Decade

**DOI:** 10.3390/jcm12113656

**Published:** 2023-05-25

**Authors:** Christopher C. M. Neumann, François Schneider, Georg Hilfenhaus, Loredana Vecchione, Christian Benzing, Jana Ihlow, Uli Fehrenbach, Thomas Malinka, Ulrich Keilholz, Sebastian Stintzing, Uwe Pelzer

**Affiliations:** 1 Department of Hematology, Oncology and Tumor Immunology, Charité-Universitätsmedizin Berlin, Freie Universität Berlin, Humboldt-Universität zu Berlin, Berlin Institute of Health, 10117 Berlin, Germany; 2 Department of Surgery|CCM|CVK, Charité-Universitätsmedizin Berlin, Freie Universität Berlin, Humboldt-Universität zu Berlin, Berlin Institute of Health, 10117 Berlin, Germanythomas.malinka@charite.de (T.M.); 3 Department of Pathology, Charité-Universitätsmedizin Berlin, Freie Universität Berlin, Humboldt-Universität zu Berlin, Berlin Institute of Health, 10117 Berlin, Germany; 4 Department of Radiology, Charité-Universitätsmedizin Berlin, Freie Universität Berlin, Humboldt-Universität zu Berlin, Berlin Institute of Health, 10117 Berlin, Germany; 5 Charité Comprehensive Cancer Center, Charité-Universitätsmedizin Berlin, 10117 Berlin, Germany

**Keywords:** pancreatic cancer, smoking, body mass index, hypertension, diabetes, kidney dysfunction, insulin-therapy, pancreatic enzyme replacement therapy

## Abstract

In addition to being risk factors for pancreatic cancer, parameters such as smoking, diabetes, or obesity might also act as potential prognostic factors for the survival of patients initially diagnosed with pancreatic cancer. By implementing one of the largest retrospective study cohorts of 2323 pancreatic adenocarcinoma (PDAC) patients treated at a single high-volume center, potential prognostic factors for survival were evaluated on the basis of 863 cases. Since parameters such as smoking, obesity, diabetes, and hypertension can cause severe chronic kidney dysfunction, the glomerular filtration rate was also considered. In the univariate analyses, albumin (*p* < 0.001), active smoking (*p* = 0.024), BMI (*p* = 0.018), and GFR (*p* = 0.002) were identified as metabolic prognostic markers for overall survival. In multivariate analyses, albumin (*p* < 0.001) and chronic kidney disease stage 2 (GFR < 90 mL/min/1.37 m^2^; *p* = 0.042) were identified as independent metabolic prognostic markers for survival. Smoking presented a nearly statistically significant independent prognostic factor for survival with a *p*-value of 0.052. In summary, low BMI, status of active smoking, and reduced kidney function at the time of diagnosis were associated with lower overall survival. No prognostic association could be observed for presence of diabetes or hypertension.

## 1. Introduction

Pancreatic cancer represents one of the most lethal types of cancer with a five-year survival rate below 5% including resected, locally-advanced, and metastasized patients [1]. For patients who underwent resection and were able to receive adjuvant chemotherapy (mFOLFIRINOX), the 5-year survival rate was reported to be as high as 43.2% [2]. Moreover, pancreatic cancer is predicted to be the second most common cause of cancer-related death by 2030 [3], gaining progressively more focus. The increasing trend can partly be explained by a demographically aging population with the median age at diagnosis being 71 [3,4]. There are crucial lifestyle risk factors that lead to a higher risk for suffering from pancreatic cancer. These include smoking, excess alcohol, and a high fat diet as well as parameters of the metabolic syndrome [3,5,6]. Once diagnosed with pancreatic cancer, however, these risk factors may contribute as prognostic factors to overall survival. 

Whereas smoking reduces the overall survival of pancreatic cancer patients [7,8], the effect of obesity still remains controversial [9,10]. Low body weight, on the other hand, is known to correlate to patients’ (pts) outcome in the context of tumor cachexia [11]. In addition to many other factors, such as inflammation and protein catabolism, exocrine and endocrine pancreas insufficiency can contribute to cachexia [12]. Whether or not Diabetes mellitus itself represents a prognostic factor has not yet been fully elucidated [13,14]. Hypertension as another parameter of the metabolic syndrome is reported to not correlate to survival of pancreatic cancer pts [15]. 

Furthermore, parameters of the metabolic syndrome are known to cause chronic kidney damage [16]. In addition to being inversely correlated to age [17], kidney function decreases in people with obesity, diabetes, and chronic hypertension [16]. 

By analyzing one of the largest retrospective patient cohorts at a single high-volume center, 2323 pts, we examined the impact of smoking, body weight, endocrine and exocrine pancreas insufficiency, hypertension, and kidney dysfunction on the overall survival in pancreatic cancer patients.

## 2. Materials and Methods

We retrospectively analyzed pts diagnosed with pancreatic cancer between 2009 and 2021 at the Charité-Universitätsmedizin Berlin. Consecutive pts were prospectively documented in our cancer database. Permission for this study was granted by the Charité ethics committee (EA/071/22). For the research question to be addressed, the following project question was defined: Do metabolic parameters at the point of diagnosis present a potential prognostic value in pancreatic cancer? Criteria defined by the PICOTS structure (population, index prognostic factor, comparator prognostic factors, outcome, timing, setting) were used for the data to be included as well as excluded [18]. Strictly following the CHARMS-PF checklist (checklist for critical appraisal and data extraction for systemic reviews of prediction modelling studies), clinical data from patients was extracted from the hospital’s patient records at the Charité Berlin [19]. For the entire patient cohort to be retrospectively evaluated, prognostic guidelines published by Riley et al. were implemented [18]. The PROGRESS (PROGnosis RESearch Strategy) Framework was used to address the research questions [18]. In the first step, the specific health outcome of patients with pancreatic cancer was defined to be the overall survival. Secondly, we evaluated and identified certain metabolic parameters, as well as kidney dysfunction, as prognostic factors for overall survival. Lastly, the effect of individual metabolic parameters and kidney dysfunction were developed, validated, and examined for the prediction of overall survival.

In total, 2323 patients with pancreatic cancer were screened for various potential prognostic factors for pancreatic cancer. The entire patient cohort of 2323 patients included patients with a histologically verified pancreatic adenocarcinoma (PDAC) only. For the diagnosis to be made, PDAC was verified by two independent pathologists within the pathology department of the Charité Berlin. Invasive intraductal papillary mucinous neoplasm (IPMN), adenosquamous carcinoma, invasive mucinous cystic neoplasm (MCN), acinar cell carcinoma (ACC), pancreatic neuroendocrine tumor (pNET), squamous cell carcinoma (SCC), and others were all excluded in the analyses presented. 

The prognostic factors to be considered were smoking history, body mass index (BMI), endocrine and exocrine pancreas insufficiency, hypertension, and albumin (g/L), as well as creatinine (md/dL). We excluded pts who did not provide sufficient information on these variables (see Supplementary Figure S1). A total of 863 pts met the inclusion parameters. In order for detailed sub-group analyses to be made and to compare our cohort with previously published data, we divided pts into three groups: resected (n = 464), non-resected/non-metastasized (n = 86), and metastasized (n = 313). The division into different sub-groups was also made as the overall survival of resected, locally-advanced, and metastasized patients is known to differ [2,20]. 

Data and laboratory values were included at the time point of pancreatic cancer diagnosis prior to any kind of intervention (chemotherapy/surgery). In the analyses considering smoking, active smoking was considered only. No differentiation was made with respect to the timespan/intensity of smoking nor between non-smokers or former smokers. Diabetes was defined as the presence of one or more of the following criteria: listed diabetes in patient history, fasting blood sugar level higher than 126 (mg/dL), a random blood sugar level higher than 200 (mg/dL), or an HbA1c value above 6.5%. Patients were classified hypertensive when taking antihypertensive medication or hypertension was listed in patients’ hospital records. Oral and insulin-based anti-diabetic therapies were identified and listed. In the case of exocrine pancreatic insufficiency, patients with a pancreatic enzyme replacement therapy (PERT) were identified.

Additionally, creatinine values at the point of diagnosis were included in this study. The glomerular filtration rate (GFR) was deduced from the creatinine according to the equation of the CKD-EPI formula (Chronic Kidney Disease Epidemiology Collaboration): estimated GFR = 141 × (S_cr_/[0.9 for male or 0.7 for female])^κ^ × (0.993)^age^, where GFR is expressed as mL/min/1.73 m^2^ of body surface area, S_cr_ is the serum creatinine expressed in mg/dL, and κ represents a sex and age dependent variable [21].

Whenever the percentage of the missing date of patient characteristics exceeded 5% (see Table 1; ECOG 37.3% and unknown tumor location 14.9%), a multiple imputation of the missing values was performed. As ECOG was an ordinal and tumor location a nominal rating scale, missing values were not imputed/replaced by median or mean values. Rather, we implemented the missing data by replacing them with an observed response from similar values according to the Hot-Deck-Imputation method [22]. The supplemented number of cases was then determined using random sampling [22].

It should be noted that there are more factors of the metabolic syndrome that are not being considered in this study. These include abnormal cholesterol or triglyceride levels. As medical records in the vast majority of cases did not list cholesterol or triglycerides abnormalities, these aspects could not be considered as prognostic markers. 

The primary endpoint of this study was defined as the overall survival (OS). The OS reflected the timespan from the date of the histologically verified diagnosis up until the date of the last follow-up or death from cancer. In order to examine the effect of single or multiple potential prognostic parameters on median OS (mOS), univariate and multivariate analyses were conducted. Optimized cut-off values for each parameter were identified by time-dependent receiver operating characteristic (ROC) curves based on the Youden method. Nearest-neighbor matching analyses were performed in this study to examine the effect of Insulin and PERT for any outcome bias to be reduced.

Kaplan–Meier plots were constructed to determine the mOS for potential prognostic factors for pancreatic cancer. Kaplan–Meier methodology and log-rank tests were implemented to compare survival curves for each group. Whenever *p*-values were less than 0.05, values were defined to be statistically significant. Cox regression models were presented as hazard ratios (HR) and were associated with a 95% confidence interval (CI).

## 3. Results

From the original patient cohort of 2323 patients diagnosed with pancreatic cancer at the Charité-Universitätsmedizin Berlin, 863 patients with available information regarding smoking status, body mass index (BMI), endocrine and exocrine pancreatic insufficiency, hypertension, serum creatinine, and albumin levels were identified and used for the following analyses. 

### 3.1. Patient Characteristics

In the study cohort, 863 pts were identified (see Table 1). The median age was 66 years with a range of 28 to 94 years. A total of 43.7% of all patients were female (377 cases) and 56.3% were male (486 cases). Most patients had an ECOG lower than two (48.4% vs. 14.3%). In 37.3% of all cases, the ECOG status was not known. A tola of 464 pts were resected (53.7%), 86 patients (10.0%) were locally advanced, and 313 pts (36.3%) metastasized. Regarding the tumor location, the majority of the tumors were located in the pancreatic head (494 pts, 57.3%). In 78 pts, the tumor was situated in the body of the pancreas and in 117 pts (13.6%) it was located in the pancreatic tail. 

Regarding smoking, 25.0% of all pts were active smokers at the time of initial diagnosis, whereas 34.4% were not active smokers. In 351 pts (40.6%), the smoking status was unknown. The majority of pts had a body mass index (BMI) greater or equal to 22 (637 pts, 73.8%) compared to those with less than 22 (226 pts, 26.2%). Moreover, 464 pts (53.7%) were resected with an R0 resection in 35.2% of the cases, R1 resection in 16.1%, and R2 resection in 0.2%. Of those patients undergoing surgical resection, 87.9% of post-operative tumor-board recommendations were in favor of an adjuvant chemotherapy (see Supplementary Table S1). Patients without recommendation for an adjuvant chemotherapy were—at time of board consultation—not fit enough, not willing to receive chemotherapy, or had complications with no expectation to receive adjuvant treatment within 12 weeks post-operatively. A total of 399 pts (46.3%) received a palliative treatment (chemotherapy or best supportive care).

### 3.2. Determination of Cut-Off Values

Optimized cut-off values for CA19-9, albumin, BMI, and GFR were identified via time-dependent receiver operating characteristic (ROC) curves based on the Youden method. For CA19-9, albumin and BMI cut-off values of 300 kU/L, 33 g/L and 22 kg/m^2^ were identified, respectively. Moreover, the cut-off value for the GFR was 90 mL/min/1.37 m^2^. 

### 3.3. Univariate and Multivariate Analyses of Patient Cohort

In the univariate analysis, advanced age (*p*< 0.001), male sex (*p* < 0.001), advanced tumor stage (*p* < 0.001), localization (head vs. body, *p* = 0.001), elevated CA19-9 values (*p* < 0.001), higher ECOG status (*p* < 0.001), decreased albumin levels (*p* < 0.001), active smoking (*p* < 0.001), lower BMI (*p* = 0.018), and chronic kidney disease stage (*p* = 0.002) correlated with a shorter overall survival. 

In multivariate analyses, advanced age (*p*< 0.001), gender (*p* < 0.001), tumor stage (*p* < 0.001), elevated CA19-9 values (*p* < 0.001), decreased albumin levels (*p* < 0.001), and chronic kidney disease stage 2 (GFR < 90 mL/min/1.37 m^2^; *p* = 0.042) were all identified as independent prognostic markers for survival. Smoking presented a nearly statistically significant independent prognostic factor for survival with a *p*-value of 0.052.

When implementing the Hot-Deck-Imputation method [22] for ECOG (37.3% unknown) and tumor location (14.9% unknown) in order for the missing values of patient characteristics to be adequately addressed, the univariate of the tumor location turned out to show statistical significance (head vs. body, *p* = 0.001, see Table 2 and Supplementary Table S5). In the case of multivariate analyses, however, the implementation of missing patient characteristics of ECOG and tumor localization did not change the statistical relevance of any other parameter. 

### 3.4. Correlation of Metabolic Parameters to mOS

**Figure 1:**
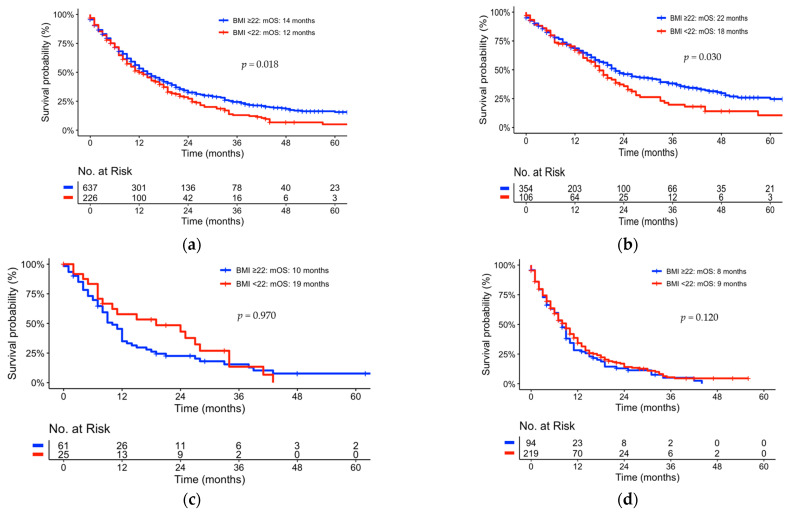
Kaplan–Meier Curves univariate comparison of sub-group analyses of BMI: (**a**) the entire patient cohort; (**b**) resected pts; (**c**) locally advanced pts; (**d**) metastasized pts; BMI—body mass index (kg/m^2^).

**Figure 2:**
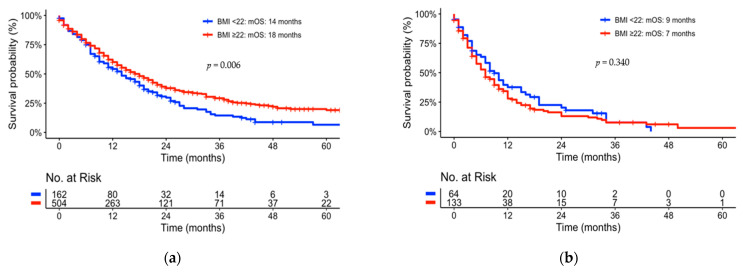
Kaplan–Meier Curves univariate comparison of sub-group analyses of BMI: (**a**) ECOG 0-1; (**b**) ECOG ≥ 2.

**Figure 3:**
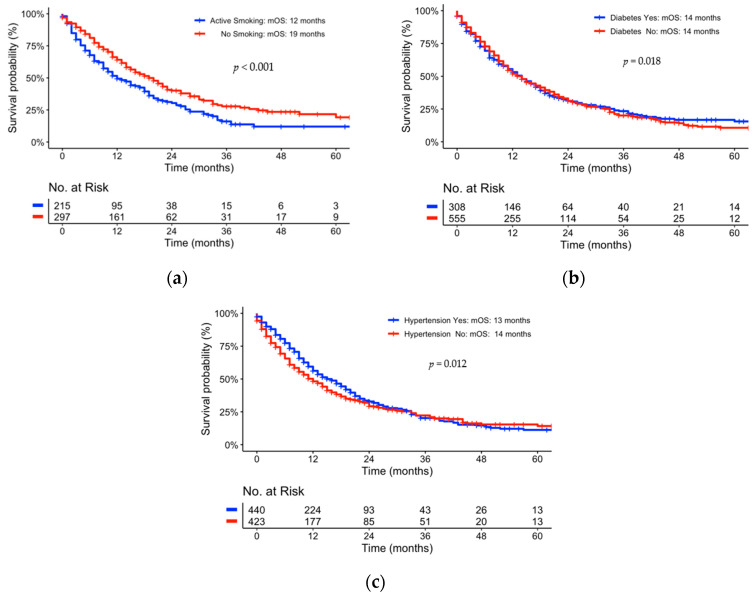
Kaplan–Meier Curves univariate comparison of mOS representing effect of: (**a**) active smoking; (**b**) diabetes; (**c**) hypertension.

With respect to individual metabolic parameters, active smoking (*p* = 0.024) as well as BMI (*p* = 0.018) showed statistical significance to overall survival in univariate analyses. Active smokers had shorter mOS than non-smokers (12 vs. 19 months, *p* < 0.001). The effect of active smoking was observed for lower ECOG status (0, 1) only (see Supplementary Tables S4 and S5). In the case of BMI, the mOS was shorter for a BMI lower than 22 kg/m^2^ (12 vs. 14 months, *p* = 0.018.) In a multivariate analysis, smoking presented a nearly statistically significant independent prognostic factor for OS with a *p*-value of 0.052 (see Table 2). BMI did not show any statistical significance in the multivariate analysis (see Table 2, Figure 1, Figure 2 and Figure 3).

When going into sub-group analyses, the correlation of higher BMI with longer survival was almost exclusively determined by the sub-group of pts undergoing resection surgery (see Figure 1 and Supplementary Table S3). However, this seems not to be associated with perioperative morbidity based on the prolonged separation of the survival curves starting 12 months after initial diagnosis.

In a further sub-group analysis, the effect of ECOG status was assessed within the group of patients with a BMI higher and lower than 22 (see Figure 2). The analysis revealed the effect on BMI as a prognostic factor for overall survival to beS only statistically significant for the patient cohort with an ECOG status of 0 and 1 (*p* = 0.006). For patients with a higher ECOG status, BMI did not show any statistical significance (*p* = 0.340).

Thus, the effect of the entire cohort presented in Figure 1a is solely an effect of resected pts with a low ECOG status (0, 1). For locally advanced or metastasized pts with pancreatic cancer, smoking and BMI at the point of diagnosis did not reflect a statistical dependence on OS. Moreover, there was no statistical significance in obese pts compared to pts with normal BMI (data not shown). Individual sub-group analyses of smoking status did not show any statistical significance of an individual tumor stage (see Supplementary Table S2 and Figure S2). Diabetes mellitus (*p* = 0.970) as well as hypertension (*p* = 0.120) did not correlate to OS and showed no statistical significance in univariate analyses (see Figure 3b,c).

### 3.5. Correlation of Kidney Function to Survival

**Figure 4:**
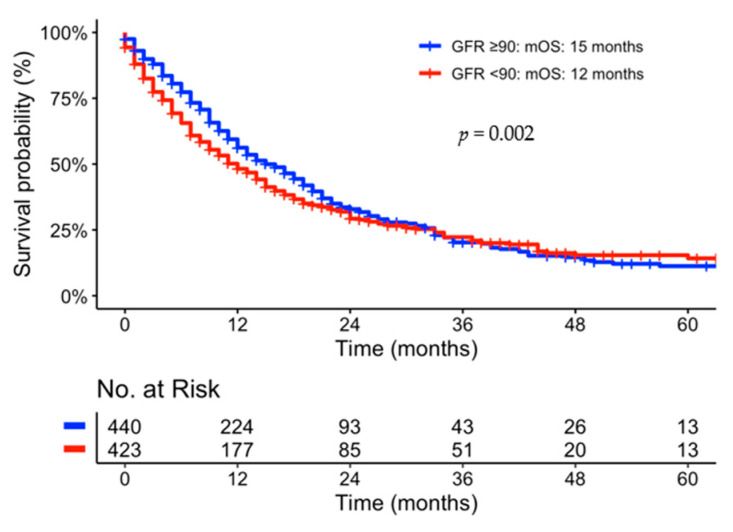
Kaplan–Meier Curve univariate comparison of mOS representing a cut-off value of GFR = 90 mL/min/1.37 m^2^.

Kidney function was measured by the glomerular filtration rate (GFR, mL/min/1.37 m^2^). The identified optimized cut-off value of 90 mL/min/1.37 m^2^ in our patient cohort represented chronic kidney disease (CKD) stage G2. Pts with a CKD G2 were correlated to a statistically significant shorter median overall survival compared to those with GFR higher than 90 mL/min/1.37 m^2^ (12 vs. 15 months, *p* = 0.002, see Figure 4). Individual sub-group analyses showed a statistical significance for locally advanced pancreatic cancer only (see Supplementary Table S4 and Figure S3). 

### 3.6. Therapy Effects of Pancreatic Endocrine and Exocrine Insufficiency

A nearest neighbor matching analysis was performed to investigate the effect of insulin therapy on the survival of pts with pancreatic cancer. A total of 112 of 863 pts received insulin therapy. The control group was carefully selected to match the treatment group with respect to clinical factors such as age, sex, tumor-stage, and patient characteristics. Amongst pts with diagnosed diabetes mellitus, the mOS was identical irrespective of whether they were treated with insulin or not (see Table 3). 

As many pts with pancreatic cancer suffer from pancreatic exocrine insufficiency [23], pancreatic enzyme replacement therapy (PERT) needs to be considered. Of the total patient cohort, 220 pts received PERT. Nearest-neighbor matching was utilized to match 220 pts of the non-PERT group to the PERT group. Even though a slightly longer median overall survival was observed for pts receiving PERT, this difference was statistically not significant (*p* = 0.12).

## 4. Discussion

This retrospective investigation analyzed potential prognostic factors that are associated with the metabolic syndrome. As the prognostic power of parameters such as body weight [9,10], diabetes [13,14], and smoking [24,25] remain controversial in pancreatic cancer, this study utilizes a patient databank of one of the largest single high throughput centers addressing these questions within the last decade. Advanced age (*p* < 0.001), sex (*p* < 0.001), tumor stage (*p* < 0.001), CA19-9 (*p* < 0.001), albumin (*p* < 0.001), and chronic kidney disease stage 2 (GFR < 90 mL/min/1.37 m^2^; *p* = 0.042) were identified as independent prognostic markers for survival in a multivariate analysis. Moreover, smoking presented a nearly statistically significant independent prognostic factor for survival with a *p*-value of 0.052. 

It is well recognized that pancreatic cancer patients with a surgically resectable tumor have a better overall survival than patients with a locally advanced or a metastazised tumor stage [2,20]. In turn, patients with a locally advanced tumor are reported to have a median survival of 9 months compared to 3 months at the metastatic stage [20]. By differentiating between resectable, locally advanced, and metastasized patients, we are more clearly differentiating the individual effects that are not being considered by many restrospective cohorts previously published (see Table 4). Moreover, certain metabolic parameters such as BMI and smoking are reported to not only affect the overall survival in patients undergoing curative resection [26,27]. BMI and smoking are also reported to have an effect for patients with a non-curative intention to treat [25,28]. Tobacco smoking represents one of the unhealthiest lifestyle risk factors. It is known to increase the risk for cardiovascular disease and cancers [29]. The risk for suffering from pancreatic cancer is increased by 75% when smoking. Once diagnosed with pancreatic cancer, smoking itself can act as a prognostic risk factor. In a meta-analysis accounting for 20 study cohorts and 15,341 pancreatic cancer pts, a longer survival was observed in non-smokers compared to smokers (HR = 1.56; 95% CI = 1.34–1.83, *p* < 0.001) [7]. In agreement with the literature, our study was able to identify active smoking as a prognostic risk factor for survival in the univariate analysis as well as a nearly statistically significant independent prognostic factor in the multivariate analysis.

Obesity is another parameter to be considered. Whereas several retrospective cohorts did not correlate survival with higher BMI values [5,10,26], one retrospective analysis of 841 pancreatic cancer pts proved a correlation with reduced overall survival (HR = 1.26, 95% CI = 0.94–1.69, *p* = 0.04). In our study cohort, higher BMI values correlated with a longer survival. Very high BMI values in the context of obesity, however, did not shorten the survival rate in our study cohort. Tumor cachexia is thought to be crucial for the survival in pancreatic cancer [12]. It is defined by significant weight loss within six months and seen in 85% of all cancer pts. In pancreatic cancer, approximately 30% of pts suffer from a cachexia-related death [37]. In the context of this study, BMI values were only considered at the time of diagnosis. Thus, the dynamics in body weight over time of the neo-adjuvant or palliative treatment might be a better prognostic risk factor for survival rather than a single-time point measurement. Of interest, further subgroup analyses of our current study revealed that the observed correlation of higher BMI with longer survival was almost exclusively determined by the subgroup of pts undergoing resection surgery with a low ECOG status. However, this seems not to be associated with perioperative morbidity based on the prolonged separation of the prolonged separation of the survival curves starting 12 months after initial diagnosis. One could hypothesize that at the point of diagnosis, pts with lower body weight might have a more metabolically consumable disease with a higher chance of micrometastases. 

One factor reflecting the nutritional state of pts is the serum albumin. As the serum albumin was found to strongly correlate to survival in univariate and multivariate analyses of our study cohort, the nutritional state of pts remains crucial. Pancreatic enzyme replacement therapy (PERT) provides one way of reducing the loss in body weight. This is particularly important for pts suffering from exocrine pancreatic insufficiency. In a previous study, PERT was statistically correlated to weight gain, and had a survival benefit [12,23,38]. In a nearest-neighbor matching analysis of our study cohort, we could not find an effect of PERT on survival. In addition to smoking and BMI, diabetes represents another risk factor for pancreatic cancer, with 85% of pts presenting with a glucose intolerance or diabetes mellitus at the point of diagnosis prior to therapeutic intervention [39]. Moreover, in vitro experiments could show hyperglycemia to stimulate pancreatic cell proliferation and chemoresistance [40]. In a very large retrospective British cohort study of 3147 pts, no difference in survival for diabetes in pancreatic cancer was observed [13]. This is in contrast to other studies in which diabetes was linked to a shorter survival [14,41]. In our study cohort, diabetes was not affecting overall survival in pancreatic cancer. Furthermore, we did not see any benefit in survival for diabetic pts treated on an insulin-based or an insulin-free antidiabetic regimen at the time of initial cancer diagnosis. 

As another factor of the metabolic syndrome, hypertension leads to cardiovascular events and an increase in pts’ morbidity and mortality. In a meta-analysis of 12 study cohorts, the use of antihypertensive medication did not correlate to the survival in pancreatic cancer [15]. In our study cohort, hypertension did not present a prognostic factor for survival, indicating the antihypertensive therapy of pancreatic cancer pts to be secondary.

Parameters of the metabolic syndrome can lead to chronic kidney damage. In a retrospective study of 961 stage IV cancer pts including solid tumors, pts with no chronic kidney disease (CKD) had a longer OS (HR = 1.43, 95% CI = 1.12–1.83, *p* < 0.001) [42]. In this study, however, there were 1347 pts with hepatobiliary and pancreatic cancer that, in turn, did not correlate to CKD. To the best of our knowledge, no previous studies considered the glomerular filtration rate (GFR) as an independent prognostic marker. Since pts are treated with potential nephrotoxic chemotherapeutics, the correlation of GFR to survival seems more than logical. In our retrospective study cohort, GFR at the time of initial diagnosis was identified as an independent prognostic factor for survival.

Nevertheless, there are limitations to the study presented. The data were analyzed retrospectively from a single center only. In addition to elevated glucose, blood pressure, and obesity, the fourth parameter of the metabolic syndrome, abnormal cholesterol, or triglycerides was not addressed in this study. Individual therapy strategies (adjuvant therapy, palliative therapy, sequential therapies) were not discussed in the manuscript. These were not sufficiently available, and, furthermore, due to the high number of patients and the assumed equal distribution of the therapy modalities, no better characterization of initial prognostic parameters would be possible. The prediction of specific therapy modalities was not the reason for this investigation. In the case of chronic kidney disease as well as smoking, no validation with an external cohort was made. In summary, this study has analyzed different parameters associated with the metabolic syndrome. Of interest, low BMI, status of active smoking, and reduced kidney function at the time of diagnosis were associated with lower overall survival. Remarkably, no prognostic association could be observed for the presence of diabetes or hypertension. After all, the median survival of these pts may too limited for long-term effects of the metabolic syndrome such as diabetes or hypertension to affect survival.

## Figures and Tables

**Table 1:** Descriptive statistics of study cohort.

Factor	Total No. (%)
No. of patients Median Age (range) years	863 66 (28–94)
Sex	
Female	377 (43.7)
Male	486 (56.3)
ECOG	
≥ 2	123 (14.3)
< 2 Unknown	418 (48.4) 322 (37.3)
Stage	
Resected	464 (53.7)
Locally advanced	86 (10.0)
Metastasized	313 (36.3)
Localization	
Head	494 (57.3)
Body	78 (9.0)
Tail Overlap Not specified	117 (13.6) 45 (5.2) 129 (14.9)
Treatment	
Resection	
R0	304 (35.2)
R1	139 (16.1)
R2	2 (0.2)
RX	19 (2.2)
Adjuvant Chemotherapy	408 (87.9)
No Adjuvant Chemotherapy	18 (3.9)
Unkown adjuv. Treatment	38 (8.2)
Palliative Treatment	399 (46.3)

**Table 2:** Univariate and multivariate logistic regression analyses for mOS (imputed data of patient characteristics ECOG and tumor localisation).

Factor	Univariate Analysis				Multivariate Analysis		
	N	HR	95% CI	*p*-Value	HR	95% CI	*p*-Value
Age (years)							
≥65	503						
<65	360	0.7	0.6–0.8	<0.001	0.7	0.5–0.8	<0.001
Sex							
Female	377						
Male	486	1.3	1.1–1.5	<0.001	1.7	1.0–2.0	<0.001
Tumor stage							
Resected	464						
Locally advanced	86	1.8	1.4–2.3	<0.001	1.7	1.3–2.8	<0.001
Metastasized	313	2.6	2.1–3.1	<0.001	2.3	1.9–3.3	<0.001
Localization							
Head	530						
Body	160	1.4	1.1–1.7	0.001	1.0	0.9–1.6	0.543
Tail	128	1.2	0.9–1.6	0.082	1.0	0.7–1.4	0.707
Overlap	45	1.6	1.1–2.3	0.012	0.9	0.6–1.5	0.855
CA19-9 (kU/L)							
≥300	444						
<300	419	0.6	0.5–0.7	<0.001	0.7	0.5–0.9	<0.001
ECOG							
>2	123						
0–1	740	0.5	0.4–0.6	<0.001	0.8	0.6–1.1	0.343
Albumin (g/L)							
≥33	345						
<33	518	1.4	1.2–1.7	<0.001	1.5	1.2–2.0	<0.001
Active Smoking							
Yes	215						
No	297	0.83	0.7–1.0	<0.001	0.8	0.6–1.0	0.052
Unknown	351						
BMI							
≥22	637						
<22	226	1.2	1.0–1.5	0.018	1.2	0.9–1.6	0.269
					0.7 ^1^	0.44–1.04 ^1^	0.078 ^1^
Diabetes							
Yes	308						
No	555	1.0	0.85–1.2	0.970	1.1	0.8–1.3	0.339
Hypertension							
Yes	507						
No	356	0.91	0.77–1.1	0.260	1.2	0.9–1.4	0.245
GFR							
≥90	440						
<90	423	1.1	0.97–1.3	0.002	1.3	1.0–1.7	0.042

^1^ Multivariate analysis for the resected group only, excluding locally advanced and metastasized pts; BMI—body mass index (kg/m^2^); GFR—glomerular filtration rate (GFR, mL/min/1.37 m^2^).

**Table 3:** Nearest-neighbor matching analyses to examine the effect of insulin therapy in the cohort of diabetes mellitus pts and the effect of PERT.

	N	Nearest Neighbor Matching	mOS	*p*-Value
DM insulin therapy				
Yes	112	112	20	
No	751	112	20	1.00
PERT				
Yes	220	220	23	
No	643	220	18	0.12

mOS—median overall survival; DM—diabetes mellitus; PERT—pancreatic enzyme replacement therapy.

**Table 4:** Summary of reported retrospective analyses of metabolic parameters as prognostic indicators in pancreatic cancer (for hypertension, no up-to-date retrospective cohort was published).

Author	Year	Country	Patient No	Parameter	Statistical Significance	Multivariate HR (95% CI)	Status
Zhang [25]	2017	China	1640	Smoking	Yes	1.02 [0.87–1.21]; *p* = NA	Mixed ^1^
Yuan [8]	2017	USA	1037	Smoking	Yes	1.40 [1.14–1.72]; *p* = 0.002	Mixed ^1^
Pelucchi [30]	2014	Italy	648	Smoking	Yes	1.37 [1.14–1.65]; *p* < 0.001	Unkown
MixBatty [31]	2008	UK	158	Smoking	Yes	1.47 [0.90–2.39]; *p* = 0.190	Mixed ^1^
Dandona [26]	2011	USA	355	Smoking	No	NA	Resected
Olson [10]	2010	USA	475	BMI	No	1.02 [0.56–1.88]; *p* = 0.940	Resected
Park [32]	2006	Korea	348	BMI	No	1.14 [0.84–1.54]; *p* = 0.169	Unkown
Dandona [26]	2011	USA	355	BMI	No	NA	Resected
Cui [27]	2022	China	329	BMI	Yes	3.21 [0.99–10.45]; *p* = 0.048	Resected
Fu [33]	2021	China	2010	BMI	Yes	0.97 [0.95–0.99]; *p* = 0.004	Mixed ^1^
Kasenda [28]	2014	Switzerland	483	BMI	Yes	1.22 [1.04–1.41]; *p* = 0.012	Non-resected
Hwang [13]	2012	USA	3147	Diabetes	Yes	0.16 [1.00–1.33]; *p* < 0.050	Mixed ^1^
Dandona [26]	2011	USA	355	Diabetes	No	NA	Resected
Balzano [34]	2016	Italy	296	Diabetes	Yes	1.45 [1.06–1.99]; *p* = NA	Resected
Hart [35]	2014	USA	488	Diabetes	No	1.06, (0.81–1.38), *p* = 0.676	Resected
Antoniak [36]	2018	USA	16,957	GFR	Yes	2.68 ^2^ [1.10–6.56]; *p* = 0.020	Resected

^1^ Mixed—includes resected, locally-advanced and metastasized PDAC; ^2^—Odds Ratio. NA—not available.

## Data Availability

The database is stored on the Charité’s own server in a legally secure manner. All data has been saved and checked in pseudonymized form. The pseudonymized data set can be requested from the project manager of the investigation (UP), if there is contractual legal protection.

## Supplementary Materials

The following supporting information can be downloaded at: https://www.mdpi.com/article/10.3390/jcm12113656/s1, Table S1: Tumor-board recommendation of adjuvant (after resection) or palliative chemotherapy; Table S2: Descriptive statistics of study cohort with respect to active smoking; Table S3: Descriptive statistics of study cohort with respect to BMI; Table S4: Descriptive statistics of study cohort with respect to GFR; Table S5: Univariate and multivariate logistic regression analyses for mOS; Figure S1: Study flow chart; Figure S2: Kaplan–Meier Curves univariate comparison of sub-group analyses of smoking; Figure S3: Kaplan–Meier Curves univariate comparison of sub-group analyses of GFR; Figure S4: Kaplan–Meier Curves univariate comparison of sub-group analyses for ECOG status 0–1; Figure S5: Kaplan–Meier Curves univariate comparison of sub-group analyses for ECOG status ≥ 2.
