# Supplementary material for: Impact of Smoking, Body Weight, Diabetes, Hypertension and Kidney Dysfunction on Survival in Pancreatic Cancer Patients—A Single Center Analysis of 2323 Patients within the Last Decade
Source: J Clin Med. 2023 May 25;12(11):3656. doi: 10.3390/jcm12113656 (PMC10253757; doi:10.3390/jcm12113656)
Supplement: Supplementary file 1 [file jcm-12-03656-s001.zip › jcm-2310923-supplementary.pdf]

Table S1. Tumor-board recommendation of adjuvant (after resection) or palliative chemotherapy.

Group	Total (%) N = 863	Chemotherapy (%) N = 797	No Chemotherapy (%) N = 28	Unknown (%) N = 38
Resected	464 (53.8)	408 (47.3)	18 (2.1)	38 (4.4)
Locally advanced	86 (10.0)	80 (9.3)	6 (0.7)	0 (0.0)
Metastasized	313 (36.2)	309 (35.8)	4 (0.4)	0 (0.0)

Table S2. Descriptive statistics of study cohort with respect to active smoking.

Factor	Overall (%) N = 512[1]	Active Smoking (%) N = 215	No Smoking (%) N = 297
**Age (years), median**	66 (28 – 94) (67 ‖ 64 ‖ 64)[2]	63 (34 – 85) (63 ‖ 62 ‖ 63)[2]	67 (28 – 89) (69 ‖ 66 ‖ 65)[2]
**Sex**			
Female	215 (42.0) (128 ‖ 19 ‖ 68)[3]	74 (14.5) (29 ‖ 10 ‖ 35)[3]	141 (27.5) (99 ‖ 9 ‖ 33)[3]
Male	297 (58.0) (164 ‖ 32 ‖ 101)[3]	141 (27.5) (67 ‖ 14 ‖ 60)[3]	156 (30.5) (97 ‖ 18 ‖ 41)[3]
**ECOG**			
≥ 2	65 (12.7) (8 ‖ 13 ‖ 44)[3]	35 (6.8) (5 ‖ 5 ‖ 25)[3]	30 (5.9) (3 ‖ 8 ‖ 19)[3]
< 2	260 (50.8) (135 ‖ 30 ‖ 95)[3]	115 (22.5) (46 ‖ 13 ‖ 56)[3]	145 (28.3) (89 ‖ 17 ‖ 39)[3]
Unknown	187 (36.5) (149 ‖ 8 ‖ 30)[3]	65 (12.7) (45 ‖ 6 ‖ 14)[3]	122 (23.8) (104 ‖ 2 ‖ 16)[3]
**Stage**			
Resected	292 (57.0)	96 (18.8)	196 (38.2)
Locally advanced	51 (10.0)	24 (4.7)	27 (5.3)
Metastasized	169 (33.0)	95 (18.6)	74 (14.4)
**Localization**			
Head	291 (56.8)	116 (22.7)	175 (34.1)
Body	50 (9.8)	20 (4.0)	30 (5.8)
Tail	70 (13.7)	30 (5.9)	40 (7.8)
Overlap	29 (5.7)	15 (2.9)	14 (2.8)
Not specified	72 (14.0)	34 (6.6)	38 (7.4)
**Treatment**			
Resection			
R0	195 (38.1)	68 (13.3)	127 (24.8)
R1	87 (16.9)	24 (4.7)	63 (12.2)
R2	2 (0.4)	0 (0.0)	2 (0.4)
RX	8 (1.6)	4 (0.8)	4 (0.8)
Palliative Treatment	220 (43.0)	119 (23.2)	101 (19.8)

[1] in the case of 351 out of 863 patients smoking status was unknown; [2] average age of sub-groups (resected ‖ locally advanced ‖ metastasized); [3] absolute number of cases in sub-groups (resected ‖ locally advanced ‖ metastasized).

Table S3. Descriptive statistics of study cohort with respect to BMI.

Factor	Overall (%) N = 863	BMI < 22 (%) N = 226	BMI ≥ 22 (%) N =637
**Age (years), median**	66 (28 – 94) (67 ‖ 68 ‖ 66)[2]	66 (28 – 85) (65 ‖ 63 ‖ 61)[2]	69 (37 – 94) (67 ‖ 68 ‖ 66)[2]

**Sex**			
Female	377 (43.7) (206‖35‖136)[3]	126 (14.6) (61‖14‖51)[3]	251 (29.1) (145‖21‖85)[3]
Male	486 (56.3) (258‖51‖177)[3]	100 (11.6) (46‖11‖43)[3]	386 (44.7) (212‖40‖134)[3]
**ECOG**			
≥ 2	123 (14.3) (13‖25‖85)[3]	34 (3.9) (3‖7‖24)[3]	89 (10.4) (10‖18‖61)[3]
< 2	418 (48.4) (203‖46‖169)[3]	113 (13.1) (55‖9‖49)[3]	305 (35.3) (148‖37‖120)[3]
Unknown	322 (37.3) (248‖15‖59)[3]	79 (9.2) (49‖9‖21)[3]	243 (28.1) (199‖6‖38)[3]
**Stage**			
Resected	464 (53.7)	107 (12.4)	357 (41.3)
Locally advanced	86 (10.0)	25 (2.9)	61 (7.1)
Metastasized	313 (36.3)	94 (10.9)	219 (25.4)
**Localization**			
Head	494 (57.3)	121 (14.1)	373 (43.2)
Body	78 (9.0)	18 (2.0)	60 (7.0)
Tail	117 (13.6)	33 (3.7)	84 (9.9)
Overlap	45 (5.2)	13 (1.5)	32 (3.7)
Not specified	129 (14.9)	41 (4.8)	88 (10.1)
**Treatment**			
Resection			
R0	304 (35.2)	71 (8.2)	233 (27.0)
R1	139 (16.1)	32 (3.7)	107 (12.4)
R2	2 (0.2)	0 (0.0)	2 (0.2)
RX	19 (2.2)	13 (1.5)	6 (0.7)
Palliative Treatment	399 (46.3)	110 (12.7)	289 (33.6)

[2] average age of sub-groups (resected‖locally advanced‖metastasized); [3] absolute number of cases in sub-groups (resected‖locally advanced‖metastasized); BMI – body mass index (kg/m^2).

Table S4. Descriptive statistics of study cohort with respect to GFR.

Factor	Overall (%) **N** = 863	GFR < 90 (%) **N** = 423	GFR ≥ 90 (%) **N** = 440
**Age (years), median**	67 (28 – 94) (67‖68‖66)[2]	70 (35 – 94) (71‖71‖69)[2]	62 (28 – 84) (63‖62‖60)[2]
**Sex**			
Female	377 (43.7) (206‖35‖136)[3]	240 (27.8) (133‖17‖90)[3]	137 (15.9) (73‖18‖46)[3]
Male	486 (56.3) (258‖51‖177)[3]	183 (21.2) (96‖25‖62)[3]	303 (35.1) (162‖26‖115)[3]
**ECOG**			
≥ 2	123 (14.3) (13‖25‖85)[3]	66 (7.7) (7‖13‖46)[3]	57 (6.6) (6‖12‖39)[3]
< 2	418 (48.4) (203‖46‖169)[3]	205 (23.8) (97‖24‖84)[3]	213 (24.6) (106‖22‖85)[3]
Unknown	322 (37.3) (248‖15‖59)[3]	152 (17.6) (125‖5‖22)[3]	170 (19.7) (123‖10‖37)[3]
**Stage**			
Resected	464 (53.7)	229 (26.5)	235 (27.2)

Locally advanced	86 (10.0)	42 (4.9)	44 (5.1)
Metastasized	313 (36.3)	152 (17.6)	161 (18.7)
**Localization**			
Head	494 (57.3)	240 (27.8)	254 (29.4)
Body	78 (9.0)	40 (4.6)	38 (4.4)
Tail	117 (13.6)	68 (7.9)	49 (5.7)
Overlap	45 (5.2)	21 (2.4)	24 (2.8)
Not specified	129 (14.9)	54 (6.3)	75 (8.7)
**Treatment**			
Resection			
R0	304 (35.2)	155 (18.0)	149 (17.2)
R1	139 (16.1)	68 (7.9)	71 (8.2)
R2	2 (0.2)	1 (0.1)	1 (0.1)
RX	19 (2.2)	7 (0.8)	12 (1.4)
Palliative Treatment	399 (46.3)	192 (22.2)	207 (24.1)

[2] average age of sub-groups (resected‖locally advanced‖metastasized); [3] absolute number of cases in sub-groups (resected‖locally advanced‖metastasized); GFR - glomerular filtration rate ((ml/min/1.37m^2).

Table S5. Univariate and multivariate logistic regression analyses for mOS.

Factor	Univariate analysis				Multivariate analysis		
	**N**	**HR**	**95% CI**	**P-value**	**HR**	**95% CI**	**P-value**
**Age (years)**							
≥ 65	503						
< 65	360	0.7	0.59 – 0.83	< 0.001	1.8	1.4 – 2.4	< 0.001
**Sex**							
Female	377						
Male	486	1.3	1.1 - 1.5	< 0.001	2.0	1.5 – 2.7	< 0.001
**Tumor stage**							
Resected	464						
Locally advanced	86	1.8	1.4 – 2.3	< 0.001	2.1	1.4 – 3.1	< 0.001
Metastasized	313	2.6	2.1 – 3.1	< 0.001	2.7	2.1 - 3.7	< 0.001
**Localization**							
Head	494						
Body	78	0.89	0.66 – 1.2	0.442	1.0	0.7 - 1.6	0.752
Tail	117	1.20	0.93 – 1.5	0.155	1.1	0.8 - 1.6	0.561
Overlap	45	1.56	1.10 – 2.2	0.016	1.2	0.8 – 2.1	0.322
Not specified	129	1.54	1.24 – 1.9	< 0.001	1.4	1.0 – 2.0	0.033
**CA19-9 (kU/l)**							
≥ 300	358						
< 300	390	0.53	0.44 – 0.63	< 0.001	0.6	0.5 – 0.8	0.0005
Unknown	115						
**ECOG**							
> 2	123						
0 - 1	418	0.53	0.42 – 0.6	< 0.001	0.6	0.4 – 0.8	0.002
Unknown	322						
**Albumin (g/l)**							
≥ 33	345						
< 33	518	1.4	1.2 – 1.7	< 0.001	1.5	0.5 – 0.8	0.0009
**Active Smoking**							
Yes	215						
No	297	0.69	0.55 – 0.86	< 0.001	0.7	0.61 - 1.0	0.056

Unknown	351						
**BMI**							
≥ 22	637						
< 22	226	1.2	1.0 – 1.5	0.018	1.2	0.89 - 1.6	0.250
					0.7[1]	0.44-1.04[1]	0.078[1]
**Diabetes**							
Yes	308						
No	555	1.0	0.85 – 1.2	0.970	1.1	0.86 - 1.5	0.386
**Hypertension**							
Yes	507						
No	356	0.91	0.77 – 1.1	0.260	1.2	0.90 – 1.5	0.227
**GFR**							
≥ 90	440						
< 90	423	1.1	0.97 – 1.3	0.002	1.4	1.0 - 1.8	0.033

[1] multivariate analysis for the resected group only, excluding locally advanced and metastasized pts; BMI – body mass index (kg/m^2); GFR – glomerular filtration rate (GFR, ml/min/1.37m^2).

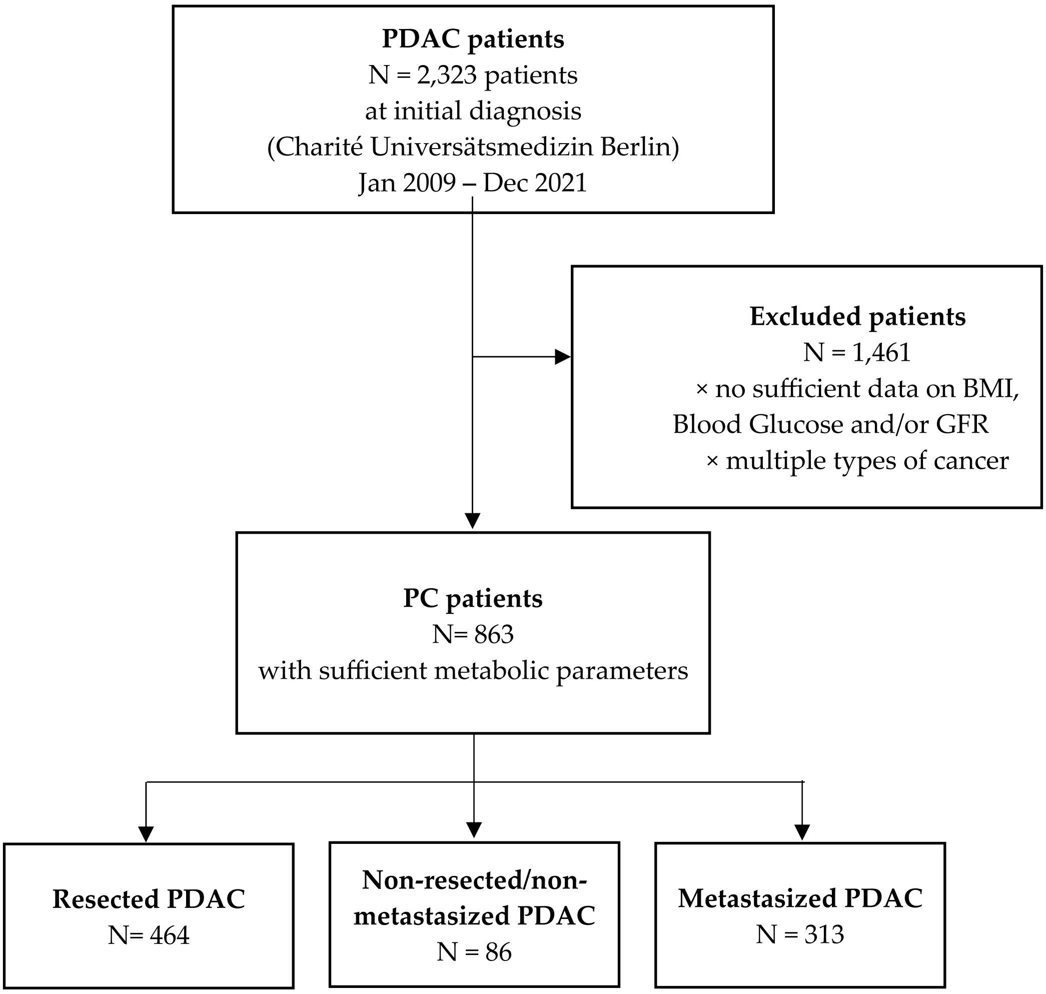

PDAC – pancreatic cancer.

Figure S1. Study flow chart.

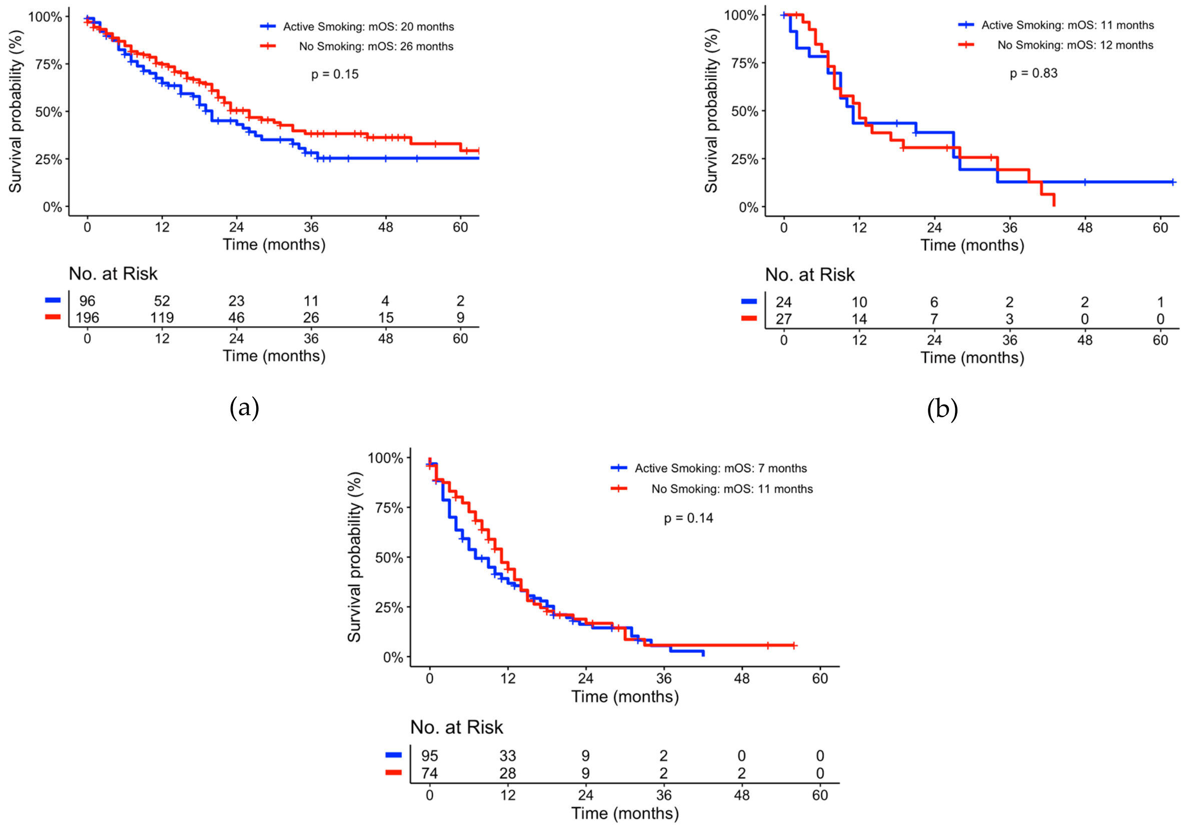

(a) (b)

(c)

Figure S2. Kaplan-Meier Curves univariate comparison of sub-group analyses of active smoking: (**a**) resected patients; (**b**) locally advanced; (**c**) metastasized patients.

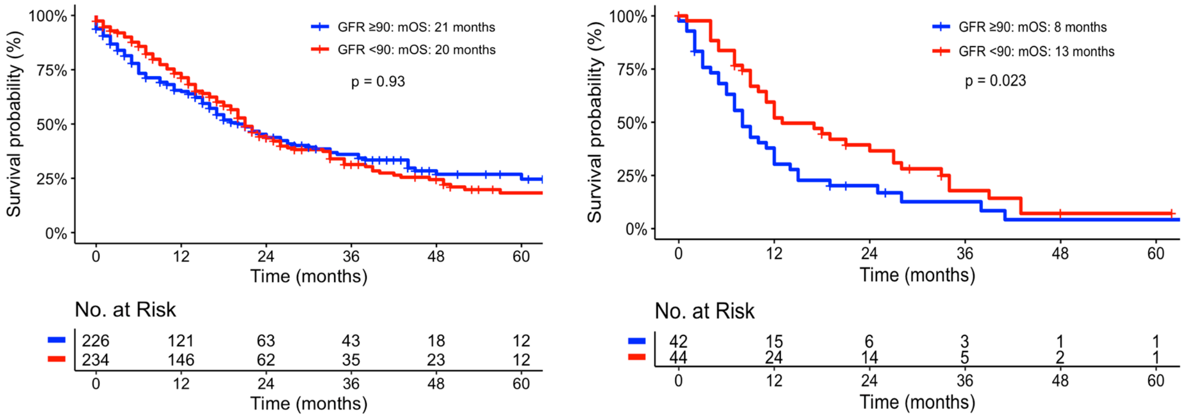

(a) (b)

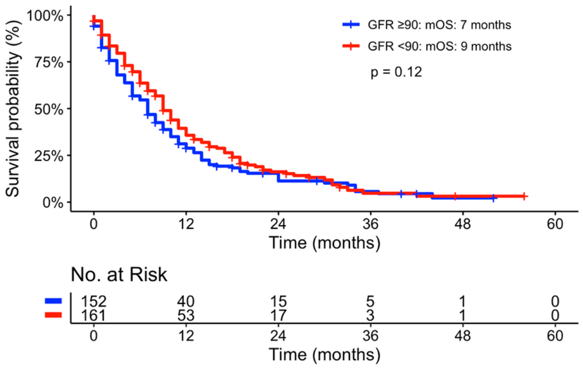

(c)

Figure S3. Kaplan-Meier Curves univariate comparison of sub-group analyses of GFR: (**a**) resected patients; (**b**) locally advanced; (**c**) metastasized patients.

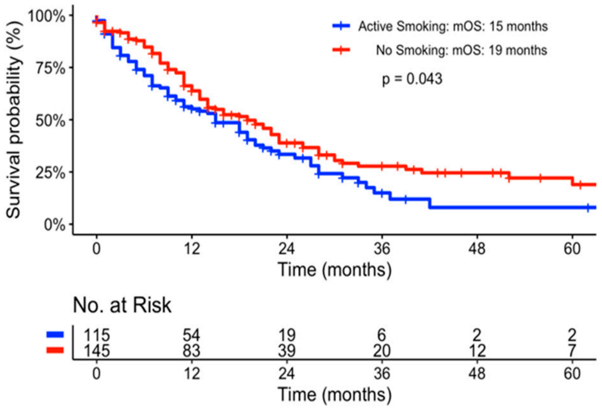

(a)

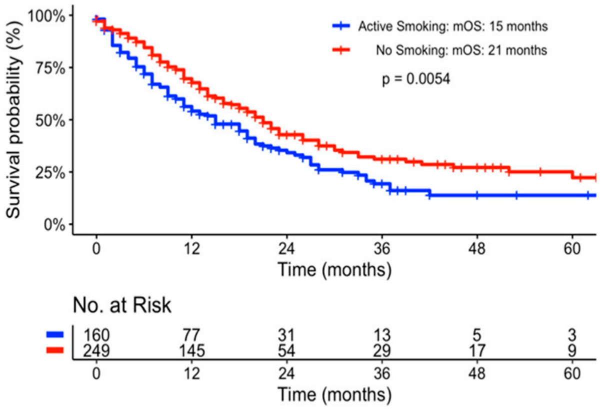

(b)

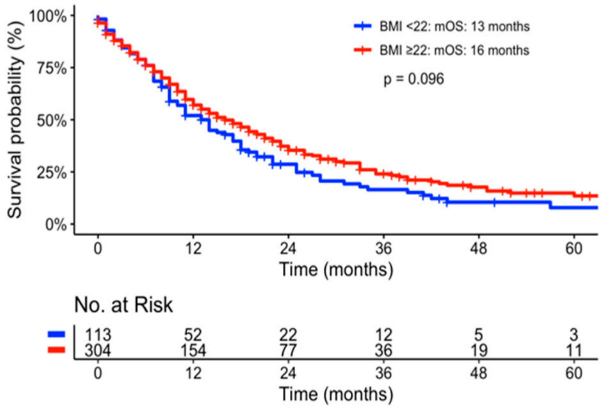

(c)

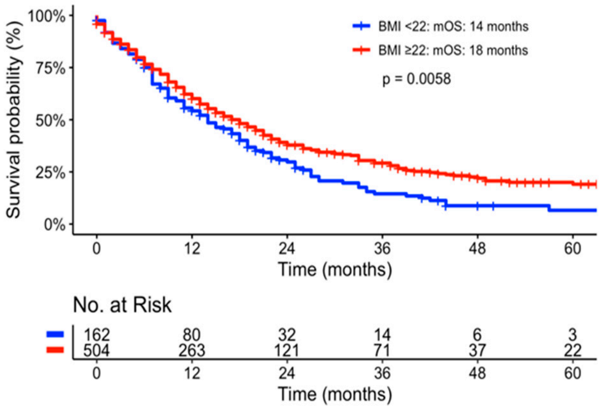

(d)

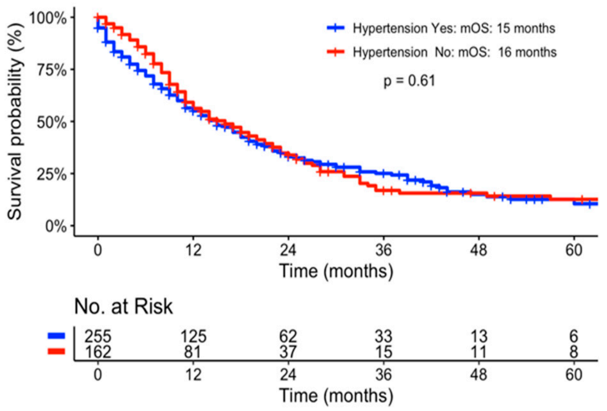

(e)

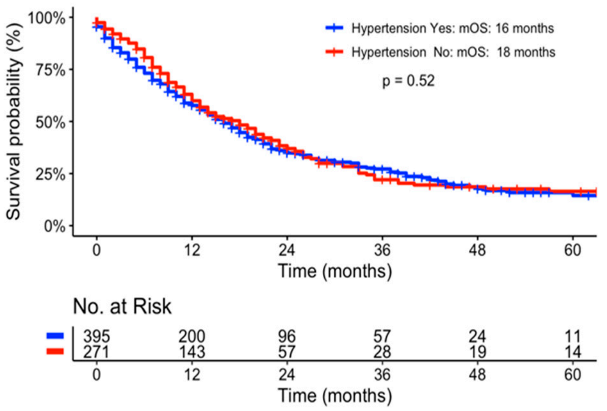

(f)

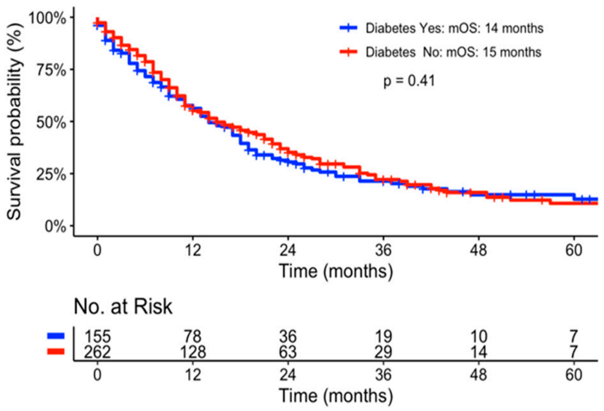

(g)

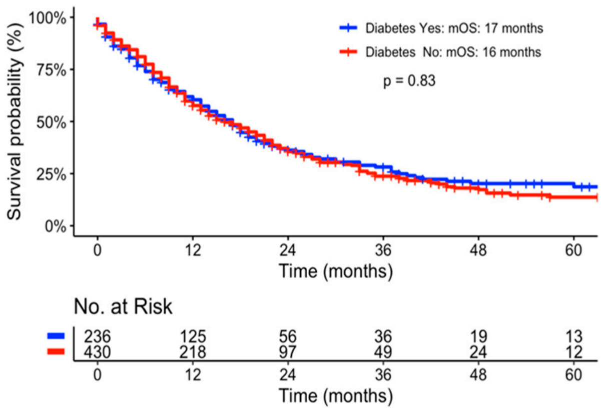

(h)

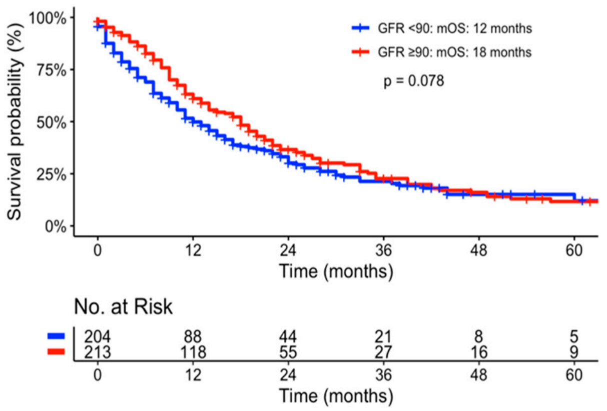

(i)

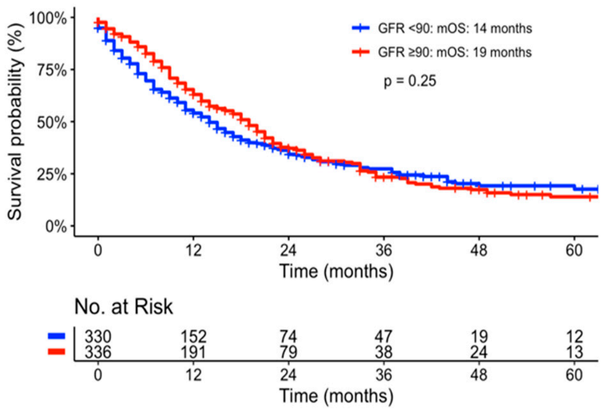

(j)

Figure S4. Kaplan-Meier Curves univariate comparison of sub-group analyses for ECOG status 0-1 **for non-imputed data** using Table S5 of: (**a**) Smoking; (**c**) BMI; (**e**) Hypertension; (**g**) Diabetes; (**i**) GFR. Kaplan-Meier Curves univariate comparison of sub-group analyses for ECOG status 0-1 **for imputed data** using Table 2 of: (**b**) Smoking; (**d**) BMI; (**f**) Hypertension; (**h**) Diabetes; (**j**) GFR.

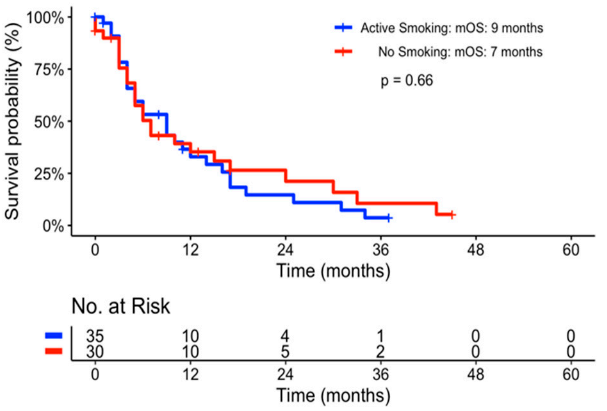
Active Smoking: mOS: 9 months
No Smoking: mOS: 7 months
p = 0.66
Survival probability (%)
Time (months)
No. at Risk
35 10 4 1 0 0
30 10 5 2 0 0

(a)

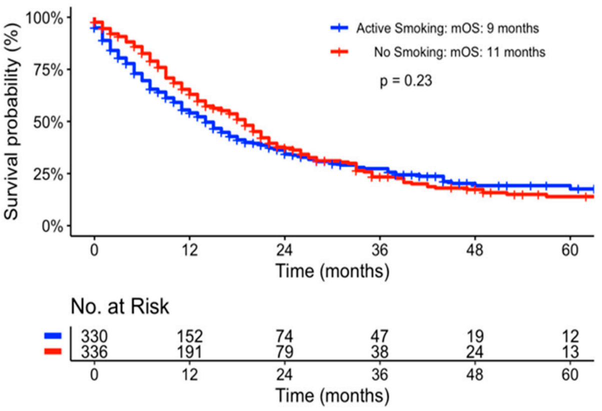
Active Smoking: mOS: 9 months
No Smoking: mOS: 11 months
p = 0.23
Survival probability (%)
Time (months)
No. at Risk
330 152 74 47 19 12
336 191 79 38 24 13

(b)

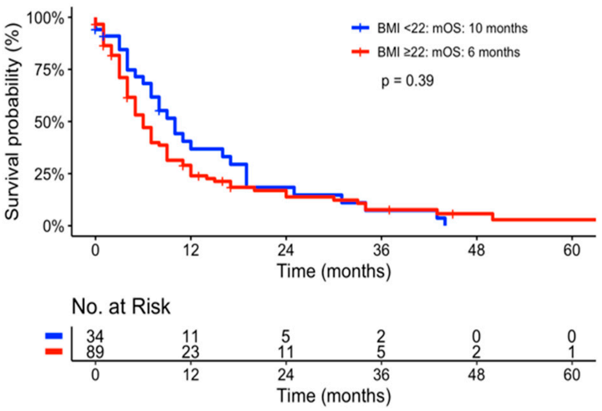
BMI <22: mOS: 10 months
BMI ≥22: mOS: 6 months
p = 0.39
Survival probability (%)
Time (months)
No. at Risk
34 11 5 2 0 0
89 23 11 5 2 1

(c)

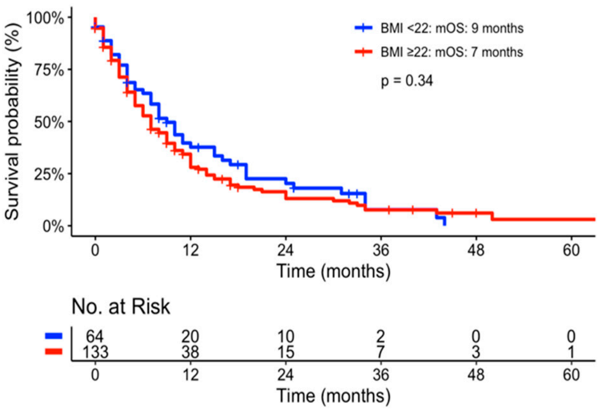
BMI <22: mOS: 9 months
BMI ≥22: mOS: 7 months
p = 0.34
Survival probability (%)
Time (months)
No. at Risk
64 20 10 2 0 0
133 38 15 7 3 1

(d)

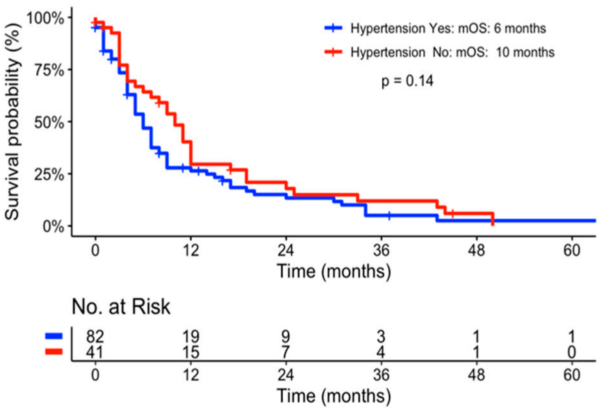
Hypertension Yes: mOS: 6 months
Hypertension No: mOS: 10 months
p = 0.14
Survival probability (%)
Time (months)
No. at Risk
82 19 9 3 1 1
41 15 7 4 1 0

(e)

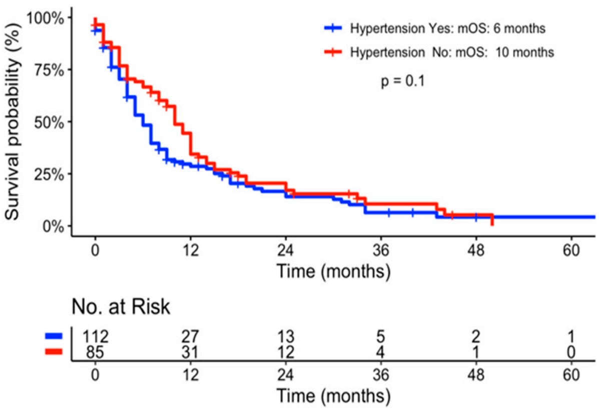
Hypertension Yes: mOS: 6 months
Hypertension No: mOS: 10 months
p = 0.1
Survival probability (%)
Time (months)
No. at Risk
112 27 13 5 2 1
85 31 12 4 1 0

(f)

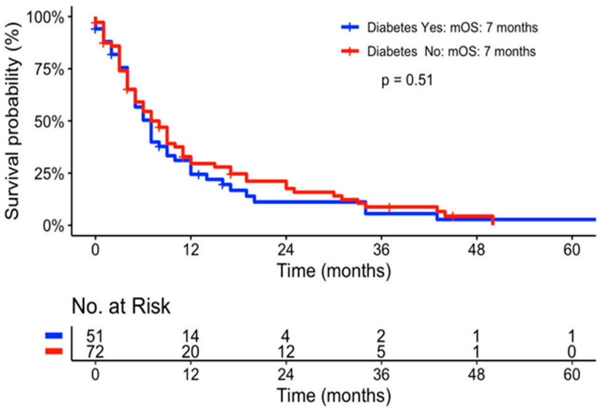
Diabetes Yes: mOS: 7 months
Diabetes No: mOS: 7 months
p = 0.51
Survival probability (%)
Time (months)
No. at Risk
51 14 4 2 1 1
72 20 12 5 1 0

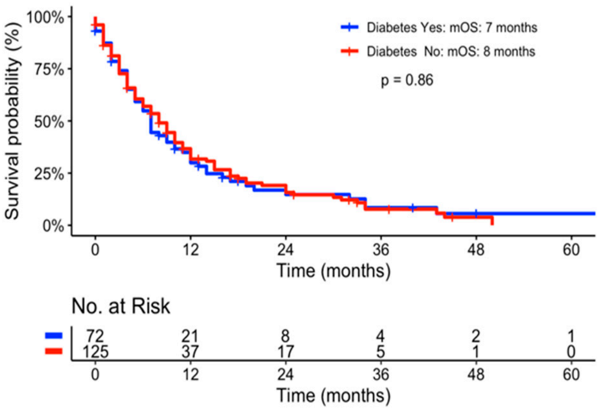
Diabetes Yes: mOS: 7 months
Diabetes No: mOS: 8 months
p = 0.86
Survival probability (%)
Time (months)
No. at Risk
72 21 8 4 2 1
125 37 17 5 1 0

(g) (h)

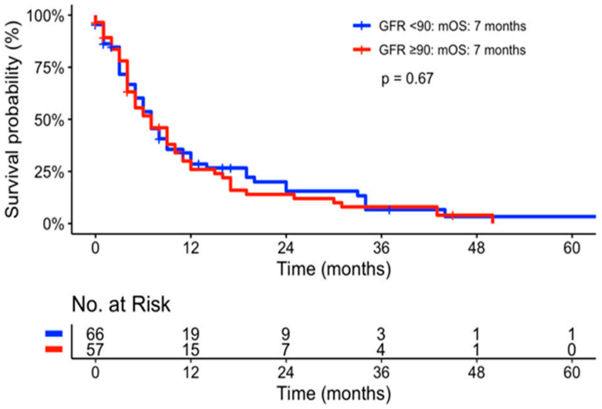

(i)

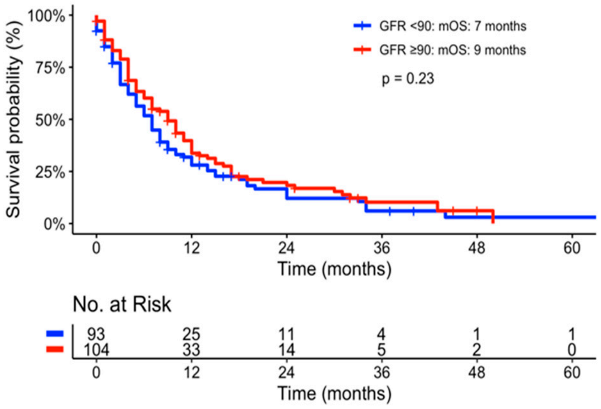

(j)

Figure S5. Kaplan-Meier Curves univariate comparison of sub-group analyses for ECOG status ≥ 2 **for non-imputed data** using Table S5 of: (**a**) Smoking; (**c**) BMI; (**e**) Hypertension; (**g**) Diabetes; (**i**) GFR. Kaplan-Meier Curves univariate comparison of sub-group analyses for ECOG status 0-1 **for imputed data** using Table 2 of: (**b**) Smoking; (**d**) BMI; (**f**) Hypertension; (**h**) Diabetes; (**j**) GFR.
